# Supplementary material for: An assessment of interobserver agreement on lesion size, morphology and clinical phenotype in cutaneous leishmaniasis caused by Leishmania aethiopica in Ethiopia
Source: NIHR Open Res. 2025 May 14;5:12. Originally published 2025 Feb 18. [Version 2] doi: 10.3310/nihropenres.13869.2 (PMC12134724; doi:10.3310/nihropenres.13869.2)
Supplement: Peer review history

# Reviewer report: Reviewer response for version 2

**DOI:** 10.3310/nihropenres.15196.r35636

**Published:** 2025-06-03

**Authors:** Lucinda Clare Fuller

**Affiliations:** 1 International Foundatioon for Dermatology, London, UK

The authors have addressed the aspects raised in the first cycle of reviews.

 The manuscript is clear and concise and represents an important contribution to progressing the process of standardisation of assessment of leishmaniasis which will be vital in future trials of treatment.

Is the work clearly and accurately presented and does it cite the current literature?

Yes

If applicable, is the statistical analysis and its interpretation appropriate?

Yes

Are all the source data underlying the results available to ensure full reproducibility?

Partly

Is the study design appropriate and is the work technically sound?

Yes

Are the conclusions drawn adequately supported by the results?

Yes

Are sufficient details of methods and analysis provided to allow replication by others?

Partly

Reviewer Expertise:

I am a dermatologist with research experience in skin neglected tropical diseases.

I confirm that I have read this submission and believe that I have an appropriate level of expertise to confirm that it is of an acceptable scientific standard.

# Reviewer report: Reviewer response for version 2

**DOI:** 10.3310/nihropenres.15196.r35637

**Published:** 2025-06-02

**Authors:** Maria Adelaida Gómez

**Affiliations:** 1 Centro Internacional de Entrenamiento e Investigaciones Medicas, Cali, Colombia

I have read the revised version and I think it covers the comments I made, and thus consider the manuscript acceptable for publication. The only one thing that came to mind is to ask if the authors received consent from the patients to include their full face picture in the publication? If not, they need to have just the lesion, not the full face, so that patients can not be identified.

Is the work clearly and accurately presented and does it cite the current literature?

Yes

If applicable, is the statistical analysis and its interpretation appropriate?

Partly

Are all the source data underlying the results available to ensure full reproducibility?

No

Is the study design appropriate and is the work technically sound?

Partly

Are the conclusions drawn adequately supported by the results?

Yes

Are sufficient details of methods and analysis provided to allow replication by others?

Partly

Reviewer Expertise:

NA

I confirm that I have read this submission and believe that I have an appropriate level of expertise to confirm that it is of an acceptable scientific standard.

# Reviewer report: Reviewer response for version 1

**DOI:** 10.3310/nihropenres.15071.r35115

**Published:** 2025-04-12

**Authors:** Maria Adelaida Gómez

**Affiliations:** 1 Centro Internacional de Entrenamiento e Investigaciones Medicas, Cali, Colombia

Major comments:

1. It is not clear at what time over the course of disease and treatment were the patients evaluated. In the discussion section one could infer patients were undergoing treatment, but it was not stated at what moment over the course of treatment were the patients evaluated. This leads me to question the relationship between the need (assessment of clinical cure) and the evaluation time point. Why was the study conducted on a single time point, instead of evaluating the disease progression over the course of treatment, for example?
2. For a study like this, images of lesions illustrating the characteristics that were more consistently identified vs. those that were not are necessary. This could also serve as a tool for reference to other clinicians experiencing similar difficulties in clinical assessment of CL (especially non-ulcerated lesions).
3. In the discussion the authors mention “There is not yet a standardized definition of severity for CL”.  WHO and PAHO have generated guidelines that allow characterization of complicated vs. uncomplicated CL, based on number of lesions, lesion size, anatomical site of lesions, among others. This classification is the basis for selection of local vs. systemic treatments. This can be used as a baseline for definition of severity.

Minor comments

1. I suggest caution with the statement of lesions developing at the site of sandfly bite, as this is not always the case (“The disease causes skin lesions on exposed parts of the body at the site of an infected sandfly bite”)
2. First paragraph of results section “mucosal CL”. In the box the classifications are either mucocutaneous or mucosal leishmaniasis. In this case, what are the authors referring to? MCL or CL? Also table 2 has “Pure mucosal”, please keep consistency in the use of terms.
3. What instrument was used for measurement of lesion size? This is relevant as the instrument used may introduce bias in the variability, or lack of, in this measurement among observers.

Is the work clearly and accurately presented and does it cite the current literature?

Yes

If applicable, is the statistical analysis and its interpretation appropriate?

Partly

Are all the source data underlying the results available to ensure full reproducibility?

No

Is the study design appropriate and is the work technically sound?

Partly

Are the conclusions drawn adequately supported by the results?

Yes

Are sufficient details of methods and analysis provided to allow replication by others?

Partly

Reviewer Expertise:

Human Cutaneous Leishmaniasis

I confirm that I have read this submission and believe that I have an appropriate level of expertise to state that I do not consider it to be of an acceptable scientific standard, for reasons outlined above.

## References

[1] : An assessment of interobserver agreement on lesion size, morphology and clinical phenotype in cutaneous leishmaniasis caused by Leishmania aethiopica in Ethiopia. *NIHR Open Research* .2025;5: 10.3310/nihropenres.13869.1 10.3310/nihropenres.13869.1 PMC1213472440469775

# Reviewer report: Reviewer response for version 1

**DOI:** 10.3310/nihropenres.15071.r34785

**Published:** 2025-02-26

**Authors:** Lucinda Clare Fuller, annika Wilder-Smith

**Affiliations:** 1 International Foundatioon for Dermatology, London, UK; 2 Faculty of Medicine, University of Zurich, Zurich, Switzerland

This manuscript reports a study nested within a larger piece of work that is referenced. It describes the  variability in clinical findings between 12 clinicians in assessing 12  known cutaneous leishmaniasis cases. The setting is a specialist hospital in Ethiopia with acknowledged experience in managing leishmaniasis and hosting the country's flagship dermatology training program. The overarching aim of the report is to highlight the need for

 1. standardised reporting structure for cutaneous leishmaniasis to support future studies developing or assessing new therapeutic approaches.

 2. Development of a standardised severity score and

 3. Development of  standardised training for clinical assessors involved in future studies to ensure consistency. 

 Whilst the training and exposure of the majority of the clinicians will be "relevant" it is not standardised and it might be worth emphasising in the discussion this fact and including a recommendation for ensuring any clinical assessors in cutaneous leishmaniasis studies have undertaken specific training on lesion analysis. It might be useful to state if any such training exists (eg on the Open WHO platform NTD channel - obviously under migration to WHO academy at the moment), is this fit for purpose ie generalisable across the globe, or is there an educational gap? 

 I assume there is no scope for inclusion of clinical photographs to illustrate the cases and phenotype characteristics. 

 Specifically small comments:

 Introduction: para 3 you mention a study in North west Ethiopia reporting common features - can you reference this?

 Methods:

 you mention a standardised proforma - is it worth showing this?

 Results

 Is it worth stating that although the clinicians included were experienced you are not able to specifically comment on cutaneous leishmaniasis training or else report on how this is covered in the local Ethiopian Dermatology Curriculum? 

 Discussion

 Whilst noting the phenotypes and clinical features being measured, might you consider highlighting those most likely to be of clinical relevance in measuring treatment impact?

 Mucosal involvement: whilst you might want to involve ENT in assisting the development of training tools to guide clinical assessors as to whether mucosal involvement is present or absent, it should be fairly straightforward to clarify with appropriate training.

 The comments are minor and I hope helpful.

Is the work clearly and accurately presented and does it cite the current literature?

Yes

If applicable, is the statistical analysis and its interpretation appropriate?

Yes

Are all the source data underlying the results available to ensure full reproducibility?

Partly

Is the study design appropriate and is the work technically sound?

Yes

Are the conclusions drawn adequately supported by the results?

Yes

Are sufficient details of methods and analysis provided to allow replication by others?

Partly

Reviewer Expertise:

I am a dermatologist with research experience in skin neglected tropical diseases.

We confirm that we have read this submission and believe that we have an appropriate level of expertise to confirm that it is of an acceptable scientific standard, however we have significant reservations, as outlined above.
